# Effectiveness of a Positive Parental Practices Training Program for Chilean Preschoolers’ Families: A Randomized Controlled Trial

**DOI:** 10.3389/fpsyg.2018.01751

**Published:** 2018-09-21

**Authors:** Paulina Rincón, Félix Cova, Sandra Saldivia, Claudio Bustos, Pamela Grandón, Carolina Inostroza, David Streiner, Vasily Bühring, Michael King

**Affiliations:** ^1^Department of Psychology, University of Concepción, Concepción, Chile; ^2^Department of Psychiatry and Mental Health, University of Concepción, Concepción, Chile; ^3^Department of Psychiatry and Behavioural Neurosciences, McMaster University, Hamilton, ON, Canada; ^4^Department of Psychiatry, University of Toronto, Toronto, ON, Canada; ^5^Division of Psychiatry, Faculty of Brain Sciences, University College London, London, United Kingdom

**Keywords:** promotion, parental practices, parenting program, universal prevention, child behavioral problems, externalizing behavior, preschool children, randomized trial

## Abstract

**Background:** Evidence for the effectiveness of parental training as a strategy for promotion of positive parental practices and prevention of child behavior problems in low and middle income countries is not conclusive. This study aims to assess the effectiveness of a universal positive parental training program designed for this context, “Día a Día” UdeC © (“Day by Day” University of Concepción), in Chilean preschoolers’ families (3–6 years old children).

**Methods:** A cluster randomized controlled trial (cRCT) was carried out in 19 preschool education centers. There were two treatment arms: 10 centers (including 178 families) were randomly assigned to the intervention group and nine centers (including 154 families) were assigned to the waiting list control condition. Intervention groups received Day by Day UdeC, a six group sessions program for parents, including two group sessions for preschool educators, focused in affective communication; daily and child-directed play; directed attention; routines and transitions; reinforcement and incentive programs; planned inattention-ignore and time out; and logical consequences. Parental practices, parental satisfaction, and presence of children behavioral problems were examined at two-time points: T1 (4 weeks before intervention) and T2 (5–6 weeks after intervention).

**Results:** Intention-to-treat analysis shows a reduction in physical punishment and an increase in parental involvement, as well as a reduction in children behavioral problems. A per-protocol analysis revealed an additional effect: increase in observed parental practices.

**Conclusion:** This cRCT provided evidence for the effectiveness of a parental training program for the promotion of positive parental practices in low and middle income countries. The observed effects of the program in decreasing physical punishment and children’s behavioral problems make it a promising strategy for prevention purposes.

**Trial Registration:** This study was registered under ISRCTN.com (ISRCTN90762146; https://doi.org/10.1186/ISRCTN90762146).

## Introduction

Promoting positive parenting practices appears to be a useful strategy to improve the welfare and psychosocial development of children, and to prevent various psychosocial and mental health difficulties (Herrman et al., 2005). A range of parenting training programs (hereinafter, parenting programs) have been shown to be effective tools for these purposes (Merry and Moor, 2015).

Given the relationship between parenting practices and the development of behavioral problems and disorders in children, several parenting programs have focused on this issue (Furlong et al., 2013; Menting et al., 2013; Forehand et al., 2014). Behavioral problems and disorders during childhood (also called externalizing problems and disorders) are considered among the commonest mental health issues in children and adolescents, resulting in considerable disturbance, especially inside the family and at school. They tend to persist and have negative consequences in several areas of development (Klahr and Burt, 2014).

Although evaluations of parenting programs have focused on the treatment of behavioral problems and disorders, research on prevention is also relevant and shows, in general, good results (Kato et al., 2015; Smedler et al., 2015). However, research has focused on efficacy (studies delivered under optimal conditions with high control from researches, for example, in University clinics) more than on effectiveness (studies conducted in real-word conditions, such as schools and primary care health centers; Streiner, 2002; Merry and Moor, 2015).

In spite of these limitations, the wide-scale implementation of parenting programs of evidence-based parenting programs has the potential to support families and favor the positive psychosocial development of children (Kato et al., 2015).

Our aim was to assess the effectiveness of a group parenting program, implemented at preschool educational centers in low and middle socioeconomic communities in Chile. The purpose of this program, called “Día a Día UdeC” © (“Day by Day” parenting program developed at University of Concepción, Chile, hereinafter Day by Day), is to strengthen positive parenting practices, specifically focusing on early intervention and prevention of behavioral problems in preschoolers. It was created based on a review of worldwide existing programs which had shown the most promising results (Kaminski et al., 2008; Gardner et al., 2009). For this research, one version of Day by Day Program assessed in a previous study was modified. This revised version is briefer than the previous one (six sessions as opposed to 10) and includes two working sessions with the educational personnel from the centers in order to make them party to the objectives of the program.

There is more evidence about the effectiveness of target parenting programs (e.g., aimed only at children at risk) rather universal parenting programs. Parenting universal programs had been less studied and they have shown little or no effect (Hiscock et al., 2008; Simkiss et al., 2013). Hiscock et al. (2008) obtained an effect size of 0.22 in the modification of hostile parental practices although these effects were not sustained over time (Bayer et al., 2010). Simkiss et al. (2013) did not see any effect of a 10-session universal training program implemented in low income families. However, the potential impact of effective universal programs, even without large effect sizes, might be relevant both in preventive and promotional aspects (Offord, 2000). For this reason, we decided to develop and assess a universal program. The promotion of positive parenting practices is potentially beneficial for all families and not only for at-risk families. Furthermore, in educational centers, universal programs are easier to implement, are better received, and the risks of stigmatization of selected families are avoided (Merry and Moor, 2015). The universal nature of the program implied special challenges, like the need to develop an intervention as brief as possible in order to encourage the widest participation by families.

Our main hypothesis was that Day by Day Program (experimental group) would result in lower levels of negative parenting practices (inconsistent and punitive practices), and higher levels of positive reinforcement parenting practices and involvement with the child than a waiting list control group. Our secondary hypotheses were that parenting training practice would result in less externalizing behaviors in the children than in the control group, and that participants in the program would show more satisfaction with their parental role and lower levels of depression.

## Materials and Methods

### Centers

Twenty preschool educational centers from the Province of Concepción, Chile, were invited to participate in the study. They all accepted the invitation, but one of them finally withdrew (before randomization). Of the 19 participating centers, five belonged to the Integra Foundation, three to the National Council of Kindergarten Schools, six were municipal schools, and five were private subsidized schools. Given the high degree of social stratification of educational centers in Chile, most of the study population were of low socioeconomic background, except in the case of private subsidized centers, where more middle-class families attend.

### Participants

**FIGURE 1:**
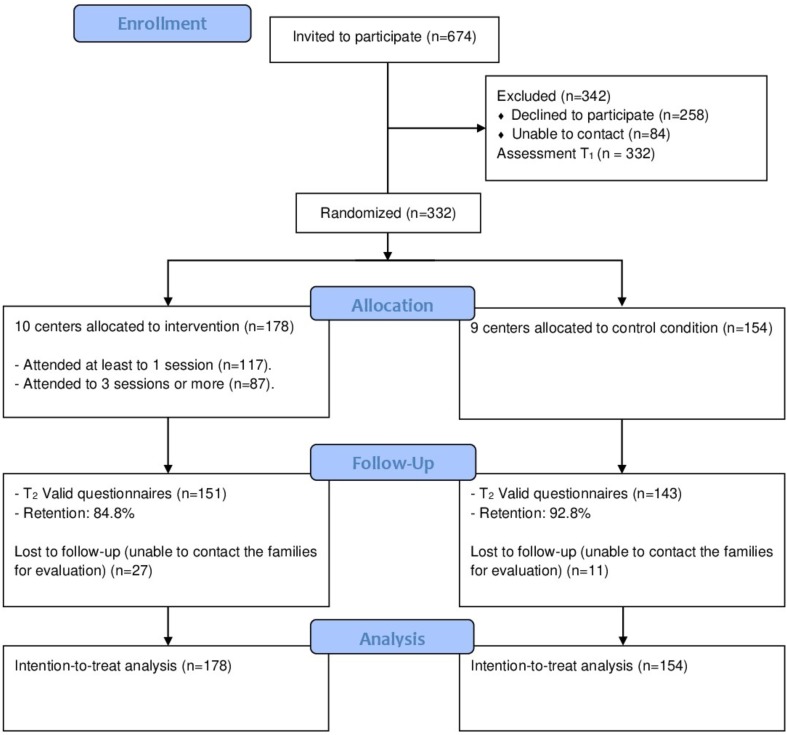
Participants flow diagram.

Families from the selected centers having children aged 3 years 0 months to 5 years 11 months were invited to participate (**Figure 1**). The invitation encouraged the participation of the mother and the father to the Day by Day Program, but only one parent or caregiver per family was considered the main participant for this effectiveness study (hereinafter “participant” or “parent”). Only this main participant was assessed and included in the analysis. Most of the participants were the children’s mothers (87%) and the remaining were other relatives (fathers, grandmothers, or sisters). In total, 674 families were invited to participate in the research, of whom 416 accepted (declined to participate, *n* = 258, a 38%). However, not everybody who accepted was able to receive the initial assessment (84 unable to contact). Since these phases were prior to randomization, only the parents or caregivers who received the initial assessment (*n* = 332) were considered as participants of this effectiveness study.

Ten centers containing 178 participants were assigned to the program, corresponding to parents of 94 boys (52.8%) and 84 girls (47.2%) with an average age of 3.7 years (SD = 1.0); the parents’ mean age was 30.6 years (SD = 6.8). Nine centers with 154 participants were assigned to the waiting list condition, corresponding to 81 boys (52.6%) and 73 girls (47.4%) with an average age of 3.9 years (SD = 1.0); their parents were 31.5 years old (SD = 6.9) on average.

The number of participants per cluster ranged from 13 to 23.

### Power and Sample Size

We sought to demonstrate an effect size at least 0.3 with an assumed intraclass correlation for clustering of 0.01, and correlation between baseline and post-test of *r* = 0.5. To achieve this at 80% power and a level of significance of 0.5%, 306 participants, 153 in each arm, were needed.

### Randomization and Masking

The randomization of the centers was performed after inviting the participants to be part of the research and conducting the baseline assessments. Thus, at the time of the pretest assessment (T_1_), participants and evaluators did not know who would participate in each group. One person from outside the research team was in charge of the entire randomization process using a ballot box and tickets. Block randomization was conducted according to the institutional affiliation of the centers. Each institution of the block, represented by numbers, was added to the ballot box. The first selected numbers, corresponding to half of each block, were assigned to the experimental group (and removed from the ballot box) and the remaining numbers to the control group. Given that three of the four blocks were odd, and that in the final balance there should be 10 experimental centers and nine control centers, the two blocks that should have an additional center assigned to the experimental group and the one that should have an additional control center were raffled.

### Training Program

Day by Day Program contains the following seven components: affective communication; daily and child-directed play; directed attention; routines and transitions; reinforcement and incentive programs; planned inattention-ignore and time out; and logical consequences. As methodological training strategies, the following are employed: participatory analysis of concepts and 19 videos showing daily interactions between one parent and the child, with different degrees of parental practice performance; role-play; rehearsal with children in the session; tasks to be applied at home; self-reports; and readings and videos to be used at home.

The implemented version of the program had six 2-h group weekly sessions. It was completely manualized and was implemented during the months of May–July 2016. It was given by psychologists with more than 5 years of psychology studies who were trained by the research team in a 40-h program (26 face-to-face hours). In order to be able to implement the program, the trained psychologists were required to undergo a performance test.

A number of measures to optimize participation were taken, such as weekly reminder calls, a day care system to allow participants to attend the center with their children, and a system of small rewards for attending (Chacko et al., 2016).

After each session, each facilitator completed a check list regarding the implementation of it, which was registered in an on-line system. The research team conducted weekly face-to-face or online therapist’s supervision. In order to assess fidelity to the program, a member of the research team made an onset observation of 10% of the sessions. Fidelity of the program, measured by adherence to program guidelines, was estimated at 84%.

### Variables and Instruments

The effect of the program was evaluated in three areas: (1) parental practices, (2) parental satisfaction, and (3) children’s behavioral problems. Pre-assessment (T_1_) was carried out 4 weeks before the beginning of the program at each center and the post-assessment (T_2_), between 5 and 6 weeks after. Evaluators were blind to the condition of the participants. Except for one instrument, all of them were answered by the adult participating person. Moreover, an observational rating scale for the assessment of the interaction of the child with the adult was used.

#### Alabama Parenting Questionnaire (APQ; Shelton et al., 1996)

This is a self-report instrument where the mother or father self-assesses the frequency with which he/she shows certain behaviors toward the child. With adaptations, it has been shown to be a suitable instrument for preschool families (Clerkin et al., 2007; de la Osa et al., 2014). It has been shown to have good inter-observer reliability in Chilean families (Cova et al., 2017). Positive reinforcement (six items), parental involvement (seven items), and disciplinary inconsistency (four items) sub-scales were used. We broadened the scope of the last sub-scale by adding three items as follows: “If you don’t get your child to obey you, finally you give up”; “One day you punish your child for doing something, but then, another day, you don’t”; and “You set clear behavior rules for your child and make him/her respect them.” We added these items on the basis that they increased Cronbach’s alpha for the now seven item subscale, had high correlations with the original scale, and improved the scale in a confirmatory analysis. Each item has five response options (1 = not much to 5 = always). The internal consistencies of each sub-scale in the initial assessment were 0.52, 0.69, and 0.75, respectively.

#### Harsh Discipline Practice List (HDPL; Flores and Herrera, 2014)

This 19-item scale measures harsh discipline behaviors, verbal maltreatment, and physical abuse and punishment. Each item has four response options (1 = never to 4 = always). On the basis of an exploratory factor analysis, four items were removed because they affected reliability or cross-loaded. Two factors were identified: (1) physical punishment (nine items) and (2) humiliating treatment (six items). The internal consistency of each factor in the initial assessment was 0.77 and 0.63, respectively.

#### Keys to Interactive Parenting Scale (KIPS; Comfort et al., 2011)

This is an observational instrument which evaluates the quality of 12 specific parental practices for caregivers of children aged 24–71 months. The interaction between the child and the caregiver in a 15–20 min play session is video recorded and afterward coded by a trained and accredited evaluator. Each parental practice is rated into five quality levels (1 = poor quality of parental practices to 5 = good quality of parental practices). The global quality of parental practices is obtained by the average score of the 12 observed parenting practices. Psychometric studies show that the instrument has acceptable internal consistency and inter-judge agreement in its application (Inostroza et al., 2014). Five percent of these assessments were coded again by an experienced evaluator and a discrepancy of only 5.4% was seen. It showed an internal consistency of α = 0.77 in the initial assessment.

#### Parental Evaluation Scale (PEP; Farkas-Klein, 2008)

This is a self-administered measure to assess satisfaction and feelings of self-efficacy with regard to parenthood. It has a Likert format of six options (0 = strongly disagree; 5 = strongly agree). The factor analysis revealed one factor. Of its 10 original items, two were eliminated, resulting in an internal consistency of α = 0.77 in the initial assessment.

#### Caregiver’s Depressive Mood Scale (Rodríguez et al., 1996)

The Caregiver’s Depressive Mood Scale from the Behavioral and Socio-Emotional Problems Inventory was used. This instrument was developed in Chile to assess the mental health of preschoolers and potential risk factors. It includes four items with two response options (Yes = 1 or No = 0) and had an internal consistency of α = 0.61 in the initial assessment.

#### Eyberg Child Behavior Inventory (ECBI; Garcia-Tornal et al., 1998; Eyberg and Pincus, 1999)

This is a 36-item instrument (each of them has seven response options from 1 = never to 7 = always), which uses parental report to assess behavioral problems in children aged between 2 and 16. It has a behavioral problem scale with seven Likert format answer options, which measure the frequency of these problems, and an intensity scale with two response options (Yes/No), which assess the extent to which each problem is a concern for the informant. Both scales had internal consistencies of α = 0.91 in the initial assessment.

### Ethical Considerations

This study was carried out in accordance with the recommendations of The International Ethical Guidelines for Biomedical Research Involving Human Subjects^1^, Research Ethics Committee of the University of Concepción, with written informed consent in accordance with the Declaration of Helsinki. The protocol was approved by the Research Ethics Committee of the University of Concepción.

### Data Analysis

In order to avoid bias due to attrition, multiple imputation procedures were used; specifically, the Markov chained equation method implemented in the mice statistics library in R.

For the main analysis comparing the experimental and control groups, a multilevel approach was used, through generalized mixed linear models, using the lme4 library in R. An approach similar to analysis of covariance (ANCOVA) was used. The dependent variable was the result in the post-test. Group membership was a fixed effect, controlling for pre-test value, sex, and age of the child. Center was considered as a nested random effect in intercept-slope models. To determine the likelihood ratio test results over imputed databases, the Wald test adapted to multiple imputation databases, called Dm, which is approximately *F* distributed, was used. The effect size was determined using the adaptation of Cohen’s *d* for ANCOVA (Cooper et al., 2009).

The analysis of the intervention results was carried out on an intention-to-treat basis (all participants assigned to the experimental condition are included regardless their attendance), as well as per-protocol, defined as attendance to three or more sessions. For this analysis, attenders for three or more sessions were compared to the participants from the control condition.

To identify possible moderators, interactions between the effects of the treatment and the pre-test values were analyzed. When the outcomes were significant, a region of significance analysis was performed, in order to identify pre-test values where treatment showed statistically significant results (Jaccard and Turrisi, 2003).

## Results

Considerable variability was seen in the attendance in the group receiving the program. Sixty one of the parents assigned to the experimental group (34%) did not participate in any of the sessions, while 117 parents attended at least one session (66%). Of these, 30 parents (17% of the initial total) participated in one or two sessions and 87 parents (49% of the initial total) participated in three or more sessions. Post-assessment was possible with 84.4% of the participants in the experimental group and 92.8% of those in the control group. Regarding KIPS, it was possible to re-evaluate 82.6% of the experimental and 89.9% of the control group. Intraclass correlation coefficients seen in the initial measurements ranged between 0.01 for parental involvement and 0.09 for concern for behavior problems.

### Effects of the Intervention

**Tables 1**, **2** show the values from the experimental and control groups for both pre and post measurements.

**Table 1:** Anova results of pre and post evaluation in experimental group.

	Pre (T_1_)		Post (T_2_)				
	Mean	*SD*	Mean	*SD*	*p*^a^	*F (df1, df2)*	est η^2^_G_^b^
Positive reinforcement	4.66	0.35	4.71	0.35	0.090	2.88 (1, 5588.26)	0.005
Parental involvement	4.39	0.52	4.52	0.46	<0.001*	15.06 (1, 745.62)	0.017
Disciplinary inconsistency	2.48	0.83	2.23	0.78	<0.001*	18.90 (1, 146.05)	0.025
Humiliating treatment	0.06	0.21	0.04	0.13	0.180	1.80 (1, 5162.47)	0.002
Physical punishment	1.01	0.50	0.77	0.40	<0.001*	66.11 (1, 881.24)	0.073
Observed parental practices	3.77	0.78	3.71	0.78	0.436	0.61 (1, 424.23)	0.002
Parental satisfaction^c^	1.61	0.95	1.46	0.92	0.010*	6.61 (1, 2082.58)	0.007
Depressed mood	0.87	0.54	0.76	0.54	0.003*	8.96 (1, 288.60)	0.010
Behavioral problems	3.16	0.85	2.76	0.85	<0.001*	65.11 (1, 972.74)	0.056
Parental concern for behavioral problems^c^	1.64	0.21	1.73	0.22	<0.001*	33.54 (1, 293.99)	0.040

^a^*Anova *p*-value. ^*b*^Squared generalized eta. ^*c*^Reverse scoring scale. ^∗^The difference between the mean scores is statistically significant *p* < 0.05*.

**Table 2:** Anova results of pre and post evaluation in control group.

	Pre (T_1_)		Post (T_2_)				
	Mean	*SD*	Mean	*SD*	p^a^	*F (df1, df2)*	est η^2^_G_^b^
Positive reinforcement	4.62	0.36	4.63	0.36	0.847	0.03 (1, 2038.39)	0.001
Parental involvement	4.35	0.53	4.35	0.52	0.949	0.00 (1, 543.78)	0.001
Disciplinary inconsistency	2.51	0.77	2.42	0.74	0.103	2.66 (1, 1676.26)	0.003
Humiliating treatment	0.07	0.14	0.06	0.12	0.430	0.62 (1, 1080.33)	0.002
Physical punishment	1.07	0.47	1.01	0.51	0.066	3.39 (1, 370.44)	0.004
Observed parental practices	3.46	0.72	3.55	0.74	0.276	1.192 (1, 567.279)	0.004
Parental satisfaction^c^	1.70	0.87	1.57	0.90	0.031*	4.70 (1, 1165.60)	0.006
Depressed mood	0.88	0.48	0.80	0.54	0.026*	5.00 (1, 259.30)	0.006
Behavioral problems	3.20	0.81	3.09	0.89	0.036*	4.38 (1, 4431.67)	0.005
Parental concern for Behavioral problems^c^	1.59	0.20	1.63	0.23	0.001*	7.02 (1, 2150.25)	0.007

^a^*Anova *p*-value. ^*b*^Squared generalized eta. ^*c*^Reverse scoring scale. ^∗^The difference between the mean scores is statistically significant *p* < 0.05*.

**Table 3** shows the analysis of the main effects. Intention-to-treat analysis showed a decrease in physical punishment practices, *d* = -0.37, *F*(1,176.4) = 20.3, *p* < 0.001; and an increase in parental involvement, *d* = 0.23, *F*(1,369.2) = 7.26, *p* = 0.007 as well as a decrease in the frequency of behavioral problems, *d* = -0.35, *F*(1,908.4) = 18.2, *p* < 0.001; and concern about them, *d* = 0.20, *F*(1,2112) = 6.88, *p* = 0.009. The per-protocol analysis showed greater effect sizes except in the last case, where it remained almost identical: physical punishment practices, *d* = -0.45, *F*(1,366,2) = 24.3, *p* < 0.001; parental involvement, *d* = 0.35, *F*(1,191.9) = 10.2, *p* = 0.002; behavioral problems, *d* = -0.40, *F*(1,2610) = 15.3, *p* < 0.001; parental concern for behavioral problems, *d* = 0.19, *F*(1,6851) = 4.04, *p* = 0.045. Per-protocol analysis also showed an increase in the observed parental practices, *d* = 0.37, *F*(1,539.1) = 8.7, *p* = 0.003.

**Table 3:** Results and effect comparison by group.

	Intention to treat			Per protocol			Adjusted effect for both groups^a^	
	*F*^b^	*p*	*d*^c^	*F*^b^	*p*	*d*^c^	Intention to treat	Per protocol
Positive reinforcement	2.700	0.101	0.164	3.220	0.073	0.217	0.605	0.292
Parental involvement	7.260	0.007*	0.227	10.200	0.002*	0.345	0.059	0.015*
Disciplinary inconsistency	2.400	0.121	-0.139	4.380	0.036	-0.227	0.607	0.218
Humiliating treatment	0.588	0.443	-0.070	1.360	0.243	-0.142	1.000	0.722
Physical punishment	20.300	<0.001*	-0.368	24.300	<0.001*	-0.447	<0.001*	<0.001*
Observed parental practices	0.582	0.446	0.084	8.700	0.003*	0.368	1.000	0.023*
Parental satisfaction^d^	0.276	0.600	-0.051	1.380	0.241	-0.130	1.000	0.722
Depressed mood	0.553	0.457	-0.067	0.603	0.438	-0.079	1.000	0.722
Behavioral problems	18.200	<0.001*	-0.348	15.300	<0.001*	-0.398	<0.001*	<0.001*
Parental concern for behavioral problems^d^	6.880	0.009*	0.202	4.040	0.045*	0.186	0.061	0.223

^a^*Multiple comparisons analysis using Bonferroni–Holm correction method. ^*b*^*F* distribution for ratio likelihood test through Wald test adapted for multiple imputations. ^*c*^Effect size. ^*d*^Reverse scoring scale. ^∗^The difference between the mean scores is statistically significant *p* < 0.05*.

Moderation analysis for intention-to-treat analysis was significant only for humiliating treatment, *F*(1,839) = 10.18, *p* = 0.001, est η^2^_G_ = 0.03 indicating that the program was effective in reducing these behaviors in those parents having higher initial levels (pre-test values on humiliating treatment greater than 0.84 standard deviations below the mean). Per-protocol, moderation was significant for positive reinforcement, *F*(1,25,587) = 5.5, *p* = 0.019, est η^2^_G_ = 0.02, and parental involvement, *F*(1,6679) = 5.081; *p* = 0.024, est η^2^_G_ = 0.02. For both variables, greater effects of the program were seen in those parents with lower initial values of positive reinforcement and parental involvement with their children (for positive reinforcement, the treatment was effective when pre-test values were lower than -0.06 standard deviations below the mean, and for parental involvement, for values lower than 0.74 standard deviations above the mean).

## Discussion

The purpose of this study was to assess the effectiveness of a brief parenting training program universally applied in families of preschool children. Although the program encouraged diverse members of the family group to participate, in practice, in our socio-cultural context, those who participate in these activities are almost only mothers.

The results are promising. At the conclusion of the program, positive changes in the expected direction in parenting practices and a decrease in the children’s behavioral problems were seen. Intention-to-treat analysis showed a decrease in humiliating treatment and physical punishment practices, and an increase in parental involvement, as well as a decrease in the frequency of behavior problems in the children and the concern about them on the part of the parents. The intention-to-treat analysis is the only approach that respects the initial randomization and, therefore, the only one that discounts the possibility that differences seen between the experimental and control groups are due to differential attrition between the groups (Kraemer, 2015). However, in this type of analysis, the magnitude of the effect size is diminished by the inclusion of people who, having said they will participate, subsequently do not. In effect, it assesses the benefit of the *offer* of training, which is the most clinically relevant where this training to be scaled up. In this study, a third of the participants did not take up the offer of training, a rate comparable to similar studies, particularly in promotional or preventative programs (Simkiss et al., 2013; Chacko et al., 2016). This, together with participants attending few sessions or irregularly, weakens the ability to detect the direct effects of the intervention.

Other factors making the detection of effects in the universal parenting training programs more difficult are the so-called floor and ceiling effects, since many parents showed suitable parenting practices prior to the intervention and many children did not show behavioral problems. Therefore, it is more difficult to demonstrate changes; thus, parenting training programs have more evident effects in those showing higher levels of problems in the initial assessment (Lundahl et al., 2008; Proctor and Brestan-Knight, 2016). We saw this in the current study, where greater effects of the program were seen in those parents with lower initial values of positive reinforcement and involvement practices with their children; importantly, in the intention-to-treat analysis, an effect of moderation was observed in relation to humiliating behaviors, indicating that the program was effective in reducing these behaviors in those parents having higher initial levels at the outset. However, the effect of floor and ceiling effects was to reduce the magnitude of the main effects of treatment.

Additionally, other effects were seen in the per-protocol analysis, specifically improved parental practices during play interactions seen in the observational data. However, given that per-protocol analyses are not balanced by the original randomization, we cannot rule out possible biases arising from participant characteristics or other factors.

This study has several limitations. First, we were unable to assess whether the favorable changes seen persisted in the longer term. Second, in spite of the universal nature of the offered program, many of the parents in the experimental group who could have benefitted from it did not participate (either refusing the invitation or not attending any of the sessions). This is particularly important for universal promotional and preventive parenting programs when some families may lack motivation to participate. In general, the main barriers to participation were time commitments and/or an incompatibility with other work or household responsibilities (Chacko et al., 2016). Considering these usual barriers, the percentage of participation was significant. We think our modification of the previous version of the Day by Day Program encouraged participation by offering fewer sessions as well as increased involvement of educational personnel in the participating centers.

A third limitation of our study is the low values of internal consistency for most of our measurements, with the exception of the Eyberg Questionnaire. Three measurements were too low (positive reinforcement, depressive mood, and humiliating treatment) and the result related to them must be interpreted with care. The Alabama Questionnaire is, however, one of the best self-report instruments to assess parental practices, and it has shown good psychometrics in the same type of population as this study (Scott et al., 2011; Cova et al., 2017).

Another limitation of this study is that its main results mainly depended on reports of the parents about their parenting practices as well as the children’s behavior. It is difficult to overcome this limitation. The assessment of the children’s behavior in a school context has been shown to have limitations and, in fact, is generally not shown to be very sensitive to the effects of parenting programs (Scott and Gardner, 2015). Thus, one of the strengths of this study was our use of an observational measure, which showed positive effects for the intervention in the per-protocol analysis. Finally, sample size was too small to assess relevance of age and sex differences in intervention’s response. Best interventions are those that specify for whom the treatment might be most effective. Future studies could elucidate any differences based on these possible moderators (Kraemer et al., 2002).

In countries like Chile, undesirable parenting behaviors, such as inappropriate treatment and physical punishment, are widely used (Vizcarra et al., 2001). Structured, replicable, evidence-based programs, based on social learning theory and encouragement of parent–child links, are a promising strategy for reducing these behaviors and need to be available in middle and low income countries (Knerr et al., 2013; Webster-Stratton and Herman, 2015).

## Conclusion

Our results demonstrate the effectiveness of this universal brief training program designed to improve parenting practices and decrease problematic behavior in children in a middle income country.

## Author Contributions

PR designed, developed, and implemented the parental training program and contributed to data collection. FC conducted study design, literature search, data interpretation, and wrote the manuscript. SS contributed to study design, program implementation, and data collection. CB performed the statistical analysis and contributed to data interpretation. PG participated in the design, development, and implementation of the program. CI conducted data collection and participated in article edition. DS participated in data interpretation and writing. VB participated in data collection and article edition. MK contributed to study design, data interpretation, and writing. All authors read and approved the final manuscript.

## Conflict of Interest Statement

The authors reports grants from CONICYT (Chilean National Commission for Scientific and Technological Research): This study was financed by the FONDEF ID14I10058. In addition, authors have a patent A-273006 issued to Universidad de Concepción: “Día a Día UdeC(c)” Parental Training Program was developed by Universidad de Concepción.

## Footnotes

1 https://cioms.ch/wp-content/uploads/2017/01/WEB-CIOMS-EthicalGuidelines.pdf
